# Yellow Fever Virus in *Aedes albopictus* Mosquitoes from Urban Green Area, São Paulo State, Brazil

**DOI:** 10.3201/eid3111.250692

**Published:** 2025-11

**Authors:** Eduardo S. Bergo, Juliana Telles de-Deus, Luis F. Mucci, Vanessa C. Helfstein, Maria de Jesus C. Nascimento, Nubia R.M.F. Rocha, Anderson de Paula, Lucy S. Villas-Boas, Camila M. Romano, Luzia M.R. Passos, Vera Lucia F. de Camargo-Neves, Karin Kirchgatter

**Affiliations:** Pasteur Institute, São Paulo, Brazil (E.S. Bergo, J. Telles-de-Deus, L.F. Mucci, V.C. Helfstein, M.J.C. Nascimento, N.R.M.F. Rocha, V.L.F. de Camargo-Neves, K. Kirchgatter); University of São Paulo, São Paulo (A. de Paula, L.S. Villas-Boas, C.M. Romano); Health Surveillance Department of the Municipality of Ribeirão Preto, São Paulo (L.M.R. Passos)

**Keywords:** vector surveillance, Aedes albopictus, yellow fever virus, outbreak, arbovirus, mosquitoes, zoonoses, Brazil, vector-borne infections, viruses

## Abstract

We detected yellow fever virus by using quantitative PCR in *Aedes albopictus* mosquitoes and isolated the virus in C6/36 cells in 4 of 18 pools, including 118 specimens collected in an urban green area in São Paulo State, Brazil. Additional monitoring to detect shifts in transmission of this species is warranted.

Yellow fever is an infectious disease caused by an RNA virus of the genus *Orthoflavivirus*, family *Flaviviridae* (*1*). Yellow fever virus (YFV) is transmitted to humans and nonhuman primates, the main vertebrate hosts, through bites of mosquitoes from genus *Aedes* in Africa and *Haemagogus* and *Sabethes* in the Americas. The sylvatic cycle occurs in both regions, where vectors, breeding and living in forests, infect nonhuman primates. Human infection is accidental (e.g., when persons enter the forest or stay at forest edges). The urban cycle, common in Africa, involves transmission between *Ae. aegypti* mosquitoes and humans. In the Americas, the last urban transmission occurred in the 1940s, when effective mass vaccination and vector-control campaigns were implemented in cities (*2*).

During 2014–2023, Brazil’s main metropolitan regions, including areas with dense, unvaccinated populations, were affected by a major yellow fever epidemic, raising concerns about disease re-urbanization (*3*). In 2017, genetic studies confirmed a new wave spread to areas outside the Amazon rainforest (*4*).

In São Paulo State, the current yellow fever epidemic (2022–2025) has reached 45 municipalities (*5*). The northwest region, which has seasonal climate and fragmented forests, reported fewer human cases and epizootics than the eastern region (*5*). YFV circulation has been documented repeatedly in 2000, 2008, 2016–2018, 2020, and 2024–2025 (*5*). In this northwest region, virus detection in secondary or potential vector species stands out, whereas in more forested regions with higher numbers of human cases and epizootics, *Haemagogus* sp. mosquitoes showed greater infectivity (*6*). We report results of an entomovirologic survey in Ribeirão Preto, São Paulo State, Brazil (≈700,000 inhabitants), conducted after epizootics occurred in nonhuman primates.

On December 25, 2024, four howler monkeys (*Alouatta caraya*) died in forest fragments on the University of São Paulo (USP) campus in Ribeirão Preto. Six days later, 2 more howler monkeys were found dead. All tested positive for YFV at the Adolfo Lutz Institute (São Paulo).

Following Brazil’s Ministry of Health guidelines, we conducted entomovirologic surveillance after confirmation of human or epizootic cases to characterize the eco-epidemiologic context. During January 7–9, 2025, four trained personnel collected adult mosquitoes by using hand nets and suction aspirators at ground level and from 10-m canopy platforms within forest fragments on the university campus during 9 AM–4 PM. We cryopreserved samples in liquid nitrogen, sent them to the Pasteur Institute (São Paulo) for morphologic identification under cold conditions, and then pooled them by species. We tested 59 female pools (197 mosquitoes from 10 species of Aedini and Sabethini tribes) (Table) for YFV RNA by quantitative reverse transcription PCR (qRT-PCR) by using a broad-range flavivirus assay (*7*) and a YFV-specific assay (*8*).

**Table:** Nonengorged adult female mosquitoes collected in entomovirologic surveillance for yellow fever virus from an urban green area (University of São Paulo, Ribeirão Preto campus), São Paulo State, Brazil, January 7–19, 2025

Species	No. mosquitoes	Pools analyzed	Positive pools
*Aedes albopictus*	118	18	4
*Ae. scapularis*	25	12	0
*Ae. serratus*	11	5	0
*Ae. terrens*	2	2	0
*Culex (Culex)* sp.	15	0	0
*Haemagogus leucocelaenus*	2	2	0
*Limatus durhami*	2	2	0
*Psorophora ferox*	3	3	0
*Sabethes albiprivus*	30	11	0
*S. glaucodaemon*	1	1	0
*S. gymnothorax*	1	1	0
*Sabethes* sp.	2	2	0
Total	212	59	4

Four pools tested positive for YFV and had high viral loads (cycle threshold [Ct] 19–22 for YFV protocol and 23–25 for pan-flavivirus protocol). We used Sanger sequencing to analyze all PCR products and confirmed YFV by using BLAST (https://blast.ncbi.nlm.nih.gov/Blast.cgi). All positive pools contained only nonengorged *Ae. albopictus* mosquitoes, collected at ground level with hand nets during January 8–9, 2025 (Table).

To further confirm the virus viability, we performed virus isolation in a Biosafety Level 2 laboratory at the USP Institute of Tropical Medicine by using *Ae. albopictus* mosquito C6/36 cells (Figure, panel A). We cultured cells at 28°C in 5% CO_2_ in Leibovitz’s L-15 medium with 5% fetal bovine serum. We filtered YFV RNA–positive pool samples (the entire bodies of the insects homogenized in Hanks’ balanced salt solution; GIBCO-BRL, https://www.thermofisher.com) through 0.22-μm membranes and inoculated them (100 μL of the filtrate) onto confluent C6/36 monolayers in 24-well plates. After 1-hour adsorption, we added 1 mL medium with 2% fetal bovine serum, 1% streptomycin, and amphotericin B. We incubated cultures 5 days and then conducted 2 passages (P2, P3). We performed daily microscopic monitoring for cytopathic effects. We tested supernatants from P1–P3 for YFV RNA by using qRT-PCR. Virus isolation succeeded in all 4 pools. Pool B3693 showed early cytopathic effects at P1 (Figure, panel B) and had a lower YFV qRT-PCR Ct than the inoculum. The other 3 pools were positive at P2 (Figure, panel C). We confirmed virus isolation by observing cytopathic effects and the decreasing Ct values during passages.

**Figure:**
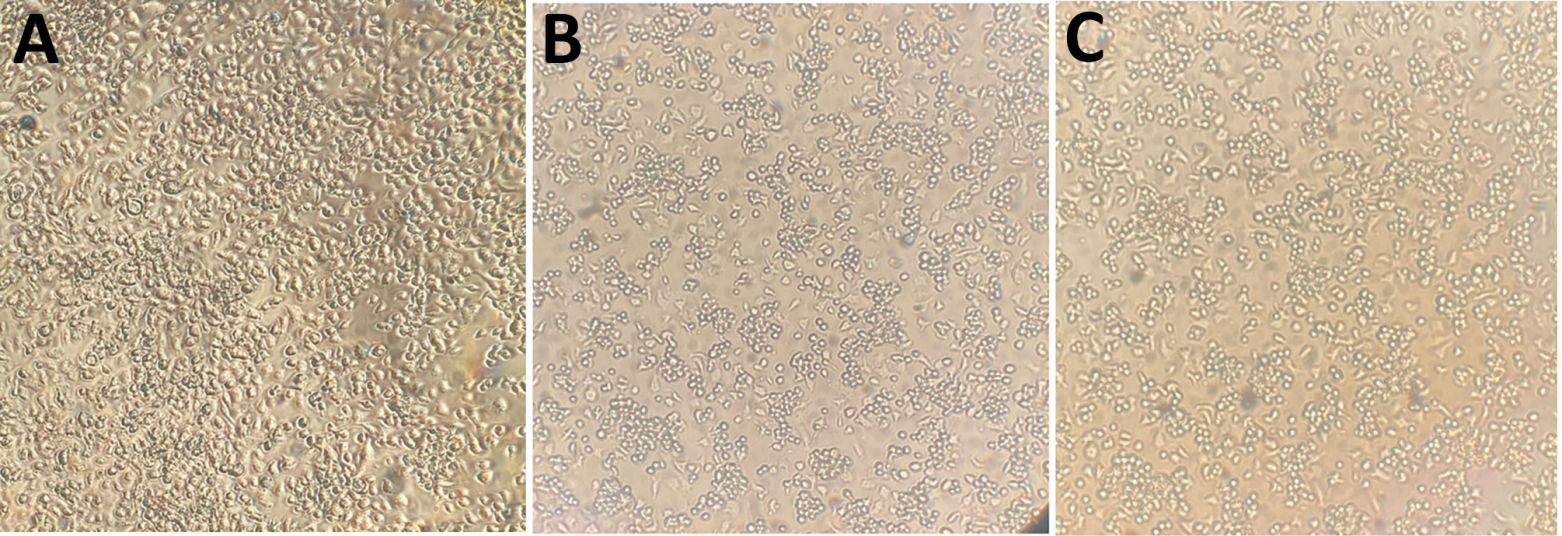
Optical microscopic analysis of C6/36 cell culture infected by yellow fever virus in *Aedes albopictus* mosquitoes from an urban green area (University of São Paulo, Ribeirão Preto campus), São Paulo State, Brazil. A) Mock (uninfected control cells). B) Yellow fever virus isolated in passage 1, five days postinfection. C) YFV isolated in passage 2, ≈10 days postinfection. Original magnification × 40.

Previous detections of YFV RNA in this species showed only low viral loads (Ct >35), and no virus was cultured (*6*). Our data suggest that *Ae. albopictus* mosquitoes played a central role in virus transmission among nonhuman primates at USP Ribeirão Preto, given its high detection rate (4 of 18 pools), abundance (55.7% of specimens), low *Haemagogus* sp. mosquito density, and no YFV found in *Sabethes albiprivus* mosquitoes (11 pools), a known secondary vector (*9*).

The confirmed vector competence of *Ae. albopictus* mosquitoes for YFV under experimental conditions (*10*), combined with our findings, highlights its potential epidemiologic role at the sylvatic–urban interface. Our findings also underscore the importance of enhancing entomological surveillance in urban green areas to detect shifts in transmission dynamics early and prevent the re-urbanization of yellow fever in Brazil.

## References

[1] Postler TS, Beer M, Blitvich BJ, Bukh J, de Lamballerie X, Drexler JF, et al. Renaming of the genus *Flavivirus* to *Orthoflavivirus* and extension of binomial species names within the family Flaviviridae. Arch Virol. 2023;168:224. 10.1007/s00705-023-05835-1 37561168

[2] Monath TP, Vasconcelos PF. Yellow fever. J Clin Virol. 2015;64:160–73. 10.1016/j.jcv.2014.08.030 25453327

[3] Giovanetti M, Pinotti F, Zanluca C, Fonseca V, Nakase T, Koishi AC, et al. Genomic epidemiology unveils the dynamics and spatial corridor behind the yellow fever virus outbreak in southern Brazil. Sci Adv. 2023;9:eadg9204. 10.1126/sciadv.adg9204 PMC10854437 37656782

[4] Andrade MS, Campos FS, Oliveira CH, Oliveira RS, Campos AAS, Almeida MAB, et al. Fast surveillance response reveals the introduction of a new yellow fever virus sub-lineage in 2021, in Minas Gerais, Brazil. Mem Inst Oswaldo Cruz. 2022;117:e220127. 10.1590/0074-02760220127 36478156 PMC9718055

[5] Saad LDC, Chiaravalloti-Neto F. Reemergence of yellow fever in the state of São Paulo: the structuring role of surveillance of epizootics in non-human primates in a one health approach. Rev Bras Epidemiol. 2024;27:e240064. 10.1590/1980-549720240064.2 39699461 PMC11654290

[6] Caleiro GS, Vilela LO, Nuevo KMB, Tubaki RM, de Menezes RMT, Mucci LF, et al. Yellow fever virus (YFV) detection in different species of culicids collected during an outbreak in southeastern Brazil, 2016–2019. Trop Med Infect Dis. 2025;10:118. 10.3390/tropicalmed10050118 40423348 PMC12115348

[7] Patel P, Landt O, Kaiser M, Faye O, Koppe T, Lass U, et al. Development of one-step quantitative reverse transcription PCR for the rapid detection of flaviviruses. Virol J. 2013;10:58. 10.1186/1743-422X-10-58 23410000 PMC3616844

[8] Domingo C, Patel P, Yillah J, Weidmann M, Méndez JA, Nakouné ER, et al. Advanced yellow fever virus genome detection in point-of-care facilities and reference laboratories. J Clin Microbiol. 2012;50:4054–60. 10.1128/JCM.01799-12 23052311 PMC3503008

[9] de Oliveira CH, Andrade MS, Campos FS. da C Cardoso J, Gonçalves-Dos-Santos ME, Oliveira RS, et al. Yellow fever virus maintained by *Sabethes* mosquitoes during the dry season in Cerrado, a semiarid region of Brazil, in 2021. Viruses. 2023;15:757. 10.3390/v15030757 PMC10058068 36992466

[10] Couto-Lima D, Madec Y, Bersot MI, Campos SS, Motta MA, Santos FBD, et al. Potential risk of re-emergence of urban transmission of Yellow Fever virus in Brazil facilitated by competent *Aedes* populations. Sci Rep. 2017;7:4848. 10.1038/s41598-017-05186-3 28687779 PMC5501812

